# A multicenter hospital-based analysis of cystic Echinococcosis in Afghanistan: Filling a National data gap

**DOI:** 10.1371/journal.pntd.0014357

**Published:** 2026-05-21

**Authors:** Mehdi Borhani, Sayed Hussain Mosawi, Sun Li, Bahetibieke Tuohetaerbaike, Saeid Fathi, Mohammad Sayed Behrad, Mohammad Shafi Fazli, Faridoon Rahmani, Ahmad Jamshid Mehrpoor, Navid Balokhil, Turyalai Hakimi, Abdulbari Omar, Mustafa Jamali, Zabihullah Adib Azizi, Hao Wen, Paul R. Torgerson

**Affiliations:** 1 State Key Laboratory of Pathogenesis, Prevention and Treatment of High Incidence Diseases in Central Asia, Xinjiang Medical University, Urumqi, China; 2 Joint Key Laboratory for International Cooperation on Major Diseases in Central Asia, Xinjiang Medical University, Urumqi, China; 3 Medical Sciences Research Center, Ghalib University, Kabul, Afghanistan; 4 Department of Parasite Vaccine Research and Production, Razi Vaccine and Serum Research Institute, Agricultural Research, Education and Extension Organization (AREEO), Karaj, Iran; 5 Department of Thoracic and Cardiovascular Surgery, Kabul University of Medical Science, Kabul, Afghanistan; 6 Seikh Zayed Hospital, Kabul, Afghanistan; 7 Thoracic Surgery Department, Ibn-Sina Chest Hospital, Kabul, Afghanistan; 8 Department of Pediatric Surgery, Maiwand teaching Hospital, Kabul University of Medical Science, Kabul, Afghanistan; 9 Department of Surgery, Istiqlal hospital, Kabul, Afghanistan; 10 The First Affiliated Hospital of Xinjiang Medical University, Department of Hepatobiliary and Hydatid Surgery, Urumqi, China; 11 Section of Epidemiology, Vetsuisse Faculty, University of Zurich, Zurich, Switzerland; Consejo Nacional de Investigaciones Cientificas y Tecnicas, Fundación Mundo Sano, ARGENTINA

## Abstract

**Background:**

Cystic Echinococcosis (CE), a neglected zoonotic disease caused by *Echinococcus granulosus*, is highly endemic in Afghanistan due to livestock-dependent livelihoods, poor sanitation, limited healthcare access, and unregulated animal practices. Despite its substantial morbidity and economic burden, national surveillance and clinical data remain scarce.

**Objective:**

This study aims to describe the clinical and demographic characteristics of surgically treated CE patients at five referral hospitals in Kabul, Afghanistan. The objective is to fill the gap of limited national-level data on CE, provide evidence for targeted clinical management, resource allocation, and inform the development of effective public health interventions to address this neglected disease.

**Methodology:**

This retrospective hospital based surgical case series analyzed 330 surgically treated CE patients admitted to five hospitals in Kabul (2021–2025). Data on demographics, geographic origin, affected organs, and clinical records were extracted from archived medical documents. Descriptive analysis prioritized epidemiological and clinical characterization.

**Results:**

Patients (6–78 years) originated from 31 provinces, with Kabul (107 cases), Faryab (35), and Balkh (24) as top sources. No significant gender predominance was observed (52.12% male, 47.87% female). Lungs were the most affected organ (86.66%), followed by the liver (11.51%); 81.31% of patients <20 years had pulmonary CE. Most cases (45.45%) were recorded in 2025, reflecting improved record retrieval.

**Discussion & conclusion:**

CE persists as a major public health challenge in Afghanistan, driven by fragmented surveillance, inadequate prevention, and unequal treatment access. The high proportion of pulmonary CE and young patients highlights unique local epidemiological patterns. Addressing CE requires a multisectoral “One Health” approach integrating surveillance, community education, veterinary interventions, and expanded diagnostic/treatment capacity to break the poverty-disease cycle.

## Author summary

Cystic echinococcosis is a serious parasitic disease that spreads from animals to humans and is very common in Afghanistan, yet almost no national data exist about this illness. We conducted a study of 330 patients treated for this disease at five major hospitals in Kabul between 2021 and 2025 to understand who is affected and how the disease presents. We found patients came from 31 provinces, with most cases affecting the lungs, especially in young people under 20 years old. Most cases were seen in 2025, likely because of better record keeping rather than more infections. Our study fills an important information gap and shows this disease remains a major public health problem in Afghanistan. We highlight the need for better disease tracking, community education, dog deworming, and safe animal handling to stop transmission and improve care for those affected.

## Introduction

Cystic Echinococcosis (CE) is a globally neglected tropical disease (NTD) caused by the larval stage of the tapeworm *Echinococcus granulosus* sensu lato. Transmitted via the fecal-oral route when humans ingest parasite eggs from contaminated food, water, or direct contact with infected dogs (definitive hosts), CE manifests as slow-growing cysts in organs, most commonly the liver (60–70%) and lungs (20–30%) [1,2]. Rare extrapulmonary/extrahepatic localizations (e.g., orbit, spine, spleen, uterus) are associated with severe morbidity, including vision loss, neurological deficits, and organ failure [3].

CE is found globally, spanning regions from China and Central Asia to the Middle East, Mediterranean countries including Southern Europe, North Africa, and Western Asia, and extending to sub-Saharan Africa and South America [4]. The World Health Organization (WHO) estimates that 1 million people worldwide live with CE, and this zoonotic infection is linked to an estimated 183,573 disability-adjusted life years (DALYs), with a range of 88,082–1,590,846 [5]. Annually, the disease imposes a $3 billion monetary burden, accounting for both treatment costs and livestock losses tied to CE [6].

The WHO Eastern Mediterranean Region (EMRO), which covers most of the Middle East and North Africa (MENA), is one of the oldest hubs for CE’s domestic transmission cycle and a recognized major hotspot for the disease [7–9]. CE remains an entrenched endemic disease in this region, driven by a complex interplay of interconnected factors.

Several high-risk animal husbandry and slaughter practices, including free-range grazing, unregulated municipal abattoirs, and widespread home slaughter, strongly favor parasite transmission. Sociocultural and behavioral factors also play a critical role: these include dietary habits such as frequent consumption of raw vegetables, large stray dog populations, and close human–dog contact influenced by local attitudes and traditions. In addition, environmental conditions support the long-term survival of the parasite eggs, while limited public awareness of transmission pathways allows the cycle to continue [9–11].

For low- and middle-income countries, CE accounts for 0.01% to 0.04% of their gross domestic product [12]. Afghanistan is a country with high endemicity of NTDs. This is primarily driven by rural livelihoods that rely heavily on livestock, along with barriers to diagnosis including poor sanitation and limited access to healthcare [13–15]. Due to poverty and remote locations, many patients with advanced disease may be unable to access treatment [16].

NTDs like CE remain largely overlooked in Afghanistan’s public health priorities. In Afghanistan, national-level data are scarce, with no functional systems in place to track cases, or monitor long-term disease trends; additionally, the animal health measures required to curb the transmission of zoonotic diseases such as CE are severely inadequate, and veterinary control measures remain minimal [17].

To address one of these gaps, given the substantial medical significance and economic impact of CE in Afghanistan, this study aims to systematically present and integrate newly collected hospital-based data on CE. The data are retrieved from surgically treated patients admitted to the surgical wards of five referral hospitals in Kabul, Afghanistan over a 5-year period (2021–2025). The primary goal of this study was to describe the clinical and demographic characteristics, including geographic origin, of surgically treated CE patients where patients from across Afghanistan seek care due to provincial healthcare gaps. This information may support targeted clinical management strategies, resource allocation, and future epidemiological research for CE in the region.

## Results

### Patient demographic characteristics

A total of 330 patients diagnosed with CE were enrolled in this study, with data collected via the archived medical documents (Table 1).

**Table 1:** Demographic, regional, institutional/ hospital, and temporal distribution of 330 CE cases (2021–2025).

*No.*	*Age*	*Gender*	*Province*	*Hospital name*	*Affected organ*	*year*
***1***	6	Female	Kabul	Maiwand pediatric	Liver	2024
***2***	7	Female	Faryab	Maiwand pediatric	Liver	2022
***3***	7	Female	Kabul	Maiwand pediatric	Liver	2023
***4***	7	Male	Kabul	Maiwand pediatric	Liver	2025
***5***	7	Female	Kabul	Maiwand pediatric	Lung	2024
***6***	7	Female	Kabul	Maiwand pediatric	Lung	2024
***7***	8	Female	Paktya	Maiwand pediatric	Liver	2024
***8***	8	Female	Kabul	Maiwand pediatric	Lung	2024
***9***	8	Female	Balkh	Maiwand pediatric	Lung	2025
***10***	9	Female	Paktya	Maiwand pediatric	Liver	2024
***11***	9	Male	Bamyan	Maiwand pediatric	Liver	2025
***12***	9	Male	Baghlan	Maiwand pediatric	Lung	2025
***13***	10	Male	Lugar	Maiwand pediatric	Liver	2025
***14***	10	Female	Kabul	^*^Kabul university of medical sciences	Lung	2022
***15***	12	Female	Kabul	Maiwand pediatrics	Liver	2024
***16***	12	Male	Parwan	Maiwand pediatrics	Liver	2023
***17***	12	Male	Kabul	Maiwand pediatrics	Liver	2023
***18***	13	Female	Sar pol	Ibn-e-Sina	Lung	2024
***19***	13	Male	Balkh	Maiwand pediatrics	Lung	2025
***20***	14	Female	Takhar	Ibn-e-Sina	Lung	2025
***21***	14	Female	Parwan	Kabul university of medical sciences	Lung	2021
***22***	14	Male	Baghlan	Ibn-e-Sina	Lung	2024
***23***	14	Male	Kabul	Ibn-e-Sina	Lung	2024
***24***	14	Male	Kabul	Ibn-e-Sina	Lung	2024
***25***	14	Male	Baghlan	Ibn-e-Sina	Lung	2024
***26***	14	Male	Helmand	Ibn-e-Sina	Lung	2025
***27***	15	Female	Ghor	Kabul university of medical sciences	Liver and Lung	2024
***28***	15	Female	Kabul	Ibn-e-Sina	Lung	2025
***29***	15	Female	Baghlan	Ibn-e-Sina	Lung	2024
***30***	15	Female	Kabul	Ibn-e-Sina	Lung	2024
***31***	15	Female	Konduz	Ibn-e-Sina	Lung	2024
***32***	15	Female	Helmand	Ibn-e-Sina	Lung	2024
***33***	15	Female	Kabul	Ibn-e-Sina	Lung	2024
***34***	15	Female	Faryab	Ibn-e-Sina	Lung	2024
***35***	15	Female	Faryab	Ibn-e-Sina	Lung	2025
***36***	15	Female	Kabul	Ibn-e-Sina	Lung	2025
***37***	15	Male	Lugar	Ibn-e-Sina	Lung	2024
***38***	15	Male	Kabul	Ibn-e-Sina	Lung	2024
***39***	15	Male	Balkh	Ibn-e-Sina	Lung	2025
***40***	15	Male	Lugar	Ibn-e-Sina	Lung	2025
***41***	15	Female	Konduz	Maiwand pediatric	Spleen	2024
***42***	16	Male	Ghor	Kabul university of medical sciences	Lung	2021
***43***	16	Female	Kabul	Maiwand pediatric	Liver	2023
***44***	16	Female	Kabul	Esteghlal Hospital	Liver	2025
***45***	16	Female	Konduz	Ibn-e-Sina	Lung	2025
***46***	16	Female	Konduz	Ibn-e-Sina	Lung	2025
***47***	16	Female	Lugar	Ibn-e-Sina	Lung	2024
***48***	16	Female	Balkh	Ibn-e-Sina	Lung	2024
***49***	16	Female	Kabul	Ibn-e-Sina	Lung	2025
***50***	16	Male	Kabul	Ibn-e-Sina	Lung	2024
***51***	16	Male	Kabul	Ibn-e-Sina	Lung	2024
***52***	16	Male	Kabul	Ibn-e-Sina	Lung	2024
***53***	16	Male	Ghazni	Ibn-e-Sina	Lung	2024
***54***	16	Male	Herat	Ibn-e-Sina	Lung	2025
***55***	16	Male	Kabul	Kabul university of medical sciences	Lung	2025
***56***	16	Male	Kabul	Kabul university of medical sciences	Lung	2021
***57***	17	Female	Kabul	Kabul university of medical sciences	Lung	2023
***58***	17	Male	Nangarhar	Ibn-e-Sina	Lung	2024
***59***	17	Male	Takhar	Ibn-e-Sina	Lung	2024
***60***	17	Male	Kapisa	Ibn-e-Sina	Lung	2025
***61***	17	Male	Kabul	Ibn-e-Sina	Lung	2025
***62***	17	Male	Samangan	Ibn-e-Sina	Lung	2025
***63***	17	Male	Ghazni	Ibn-e-Sina	Lung	2025
***64***	17	Male	Balkh	Ibn-e-Sina	Lung	2025
***65***	18	Female	Kabul	Refah Hospital	Brain	2024
***66***	18	Female	Konduz	Ibn-e-Sina	Lung	2025
***67***	18	Female	Jawzjan	Ibn-e-Sina	Lung	2024
***68***	18	Female	Konduz	Ibn-e-Sina	Lung	2024
***69***	18	Female	Takhar	Ibn-e-Sina	Lung	2025
***70***	18	Female	Ghazni	Ibn-e-Sina	Lung	2025
***71***	18	Female	Lugar	Kabul university of medical sciences	Lung	2025
***72***	18	Female	Kabul	Kabul university of medical sciences	Lung	2021
***73***	18	Male	Faryab	Ibn-e-Sina	Lung	2025
***74***	18	Male	Kabul	Ibn-e-Sina	Lung	2024
***75***	18	Male	Nangarhar	Ibn-e-Sina	Lung	2024
***76***	18	Male	Ghazni	Ibn-e-Sina	Lung	2025
***77***	18	Male	Paktya	Ibn-e-Sina	Lung	2025
***78***	18	Male	Balkh	Ibn-e-Sina	Lung	2025
***79***	18	Male	Kabul	Ibn-e-Sina	Lung	2025
***80***	18	Male	Ghazni	Ibn-e-Sina	Lung	2025
***81***	18	Male	Kabul	Ibn-e-Sina	Lung	2025
***82***	18	Male	Balkh	Ibn-e-Sina	Lung	2025
***83***	18	Male	Badakhshan	Kabul university of medical sciences	Lung	2025
***84***	18	Male	Ghazni	Kabul university of medical sciences	Lung	2022
***85***	19	Male	Kabul	Esteghlal Hospital	Liver	2025
***86***	19	Female	Sar Pol	Ibn-e-Sina	Lung	2025
***87***	19	Female	Herat	Ibn-e-Sina	Lung	2024
***88***	19	Female	Konduz	Ibn-e-Sina	Lung	2024
***89***	19	Female	Kabul	Ibn-e-Sina	Lung	2024
***90***	19	Male	Balkh	Ibn-e-Sina	Lung	2024
***91***	19	Male	Jawzjan	Ibn-e-Sina	Lung	2025
***92***	20	Female	Kabul	Kabul university of medical sciences	Liver	2023
***93***	20	Female	Lugar	Esteghlal Hospital	Liver	2025
***94***	20	Female	Kabul	Esteghlal Hospital	Liver	2025
***95***	20	Female	Balkh	Ibn-e-Sina	Lung	2024
***96***	20	Female	Jawzjan	Ibn-e-Sina	Lung	2024
***97***	20	Female	Herat	Ibn-e-Sina	Lung	2024
***98***	20	Female	Faryab	Ibn-e-Sina	Lung	2024
***99***	20	Female	Kabul	Ibn-e-Sina	Lung	2024
***100***	20	Female	Urozgan	Ibn-e-Sina	Lung	2024
***101***	20	Female	Kabul	Ibn-e-Sina	Lung	2024
***102***	20	Female	Nangarhar	Ibn-e-Sina	Lung	2024
***103***	20	Female	Balkh	Ibn-e-Sina	Lung	2024
***104***	20	Female	Ghor	Kabul university of medical sciences	Lung	2022
***105***	20	Female	Faryab	Kabul university of medical sciences	Lung	2021
***106***	20	Male	Sar pol	Ibn-e-Sina	Lung	2024
***107***	20	Male	Balkh	Ibn-e-Sina	Lung	2024
***108***	20	Male	Kunar	Ibn-e-Sina	Lung	2024
***109***	20	Male	Kabul	Ibn-e-Sina	Lung	2024
***110***	20	Male	Konduz	Ibn-e-Sina	Lung	2024
***111***	20	Male	Kabul	Ibn-e-Sina	Lung	2025
***112***	20	Male	Faryab	Ibn-e-Sina	Lung	2025
***113***	20	Male	Sar pol	Ibn-e-Sina	Lung	2025
***114***	20	Male	Kabul	Ibn-e-Sina	Lung	2025
***115***	20	Male	Sar Pol	Ibn-e-Sina	Lung	2025
***116***	20	Male	Kabul	Ibn-e-Sina	Lung	2025
***117***	20	Male	Daykundi	Ibn-e-Sina	Lung	2025
***118***	20	Male	Kabul	Ibn-e-Sina	Lung	2025
***119***	21	Female	Kabul	Kabul university of medical sciences	Liver	2022
***120***	21	Female	Faryab	Kabul university of medical sciences	Liver	2022
***121***	21	Female	Kabul	Kabul university of medical sciences	Liver	2022
***122***	21	Female	Balkh	Ibn-e-Sina	Lung	2024
***123***	21	Female	Badakhshan	Ibn-e-Sina	Lung	2024
***124***	21	Female	Kabul	Ibn-e-Sina	Lung	2024
***125***	21	Male	Balkh	Ibn-e-Sina	Lung	2024
***126***	21	Male	Konduz	Ibn-e-Sina	Lung	2024
***127***	22	Female	Ghor	Ibn-e-Sina	Lung	2024
***128***	22	Female	Kandahar	Ibn-e-Sina	Lung	2024
***129***	22	Female	Badakhshan	Ibn-e-Sina	Lung	2025
***130***	22	Male	Balkh	Ibn-e-Sina	Lung	2024
***131***	22	Male	Kabul	Ibn-e-Sina	Lung	2025
***132***	22	Male	Paktya	Ibn-e-Sina	Lung	2024
***133***	22	Male	Kabul	Ibn-e-Sina	Lung	2024
***134***	22	Male	Kabul	Ibn-e-Sina	Lung	2024
***135***	22	Male	Laghman	Ibn-e-Sina	Lung	2024
***136***	22	Male	Parwan	Ibn-e-Sina	Lung	2024
***137***	22	Male	Balkh	Ibn-e-Sina	Lung	2024
***138***	22	Male	Lugar	Ibn-e-Sina	Lung	2025
***139***	22	Male	Badakhshan	Ibn-e-Sina	Lung	2025
***140***	22	Male	Faryab	Ibn-e-Sina	Lung	2025
***141***	22	Male	Kabul	Ibn-e-Sina	Lung	2025
***142***	22	Male	Kabul	Ibn-e-Sina	Lung	2025
***143***	22	Male	Kabul	Ibn-e-Sina	Lung	2025
***144***	22	Male	Faryab	Ibn-e-Sina	Lung	2025
***145***	22	Male	Kabul	Kabul university of medical sciences	Lung	2023
***146***	22	Male	Kabul	Kabul university of medical sciences	Lung	2023
***147***	23	Male	Faryab	Esteghlal Hospital	Liver	2025
***148***	23	Male	Ghazni	Esteghlal Hospital	Liver	2025
***149***	23	Male	Ghazni	Esteghlal Hospital	Liver	2025
***150***	23	Female	Takhar	Ibn-e-Sina	Lung	2025
***151***	23	Female	Kunar	Ibn-e-Sina	Lung	2024
***152***	23	Female	Herat	Ibn-e-Sina	Lung	2024
***153***	23	Female	Takhar	Ibn-e-Sina	Lung	2024
***154***	23	Female	Faryab	Ibn-e-Sina	Lung	2025
***155***	23	Female	Kabul	Kabul university of medical sciences	Lung	2021
***156***	23	Male	Faryab	Ibn-e-Sina	Lung	2025
***157***	23	Male	Kabul	Kabul university of medical sciences	Lung	2022
***158***	24	Female	Balkh	Ibn-e-Sina	Lung	2025
***159***	24	Male	Paktya	Kabul university of medical sciences	Lung	2025
***160***	24	Male	Paktya	Kabul university of medical sciences	Lung	2025
***161***	25	Male	Nangarhar	Esteghlal Hospital	Liver	2025
***162***	25	Female	Zabul	Ibn-e-Sina	Lung	2024
***163***	25	Female	Faryab	Ibn-e-Sina	Lung	2024
***164***	25	Female	Parwan	Ibn-e-Sina	Lung	2025
***165***	25	Female	Maydan Wardak	Ibn-e-Sina	Lung	2025
***166***	25	Female	Parwan	Ibn-e-Sina	Lung	2025
***167***	25	Female	Herat	Kabul university of medical sciences	Lung	2023
***168***	25	Male	Jowzjan	Ibn-e-Sina	Lung	2024
***169***	25	Male	Faryab	Ibn-e-Sina	Lung	2025
***170***	25	Male	Konduz	Ibn-e-Sina	Lung	2025
***171***	25	Male	Faryab	Kabul university of medical sciences	Lung	2021
***172***	26	Female	Parwan	Esteghlal Hospital	Liver	2025
***173***	26	Female	Kabul	Ibn-e-Sina	Lung	2024
***174***	26	Female	Baghlan	Ibn-e-Sina	Lung	2024
***175***	26	Female	Jawzjan	Ibn-e-Sina	Lung	2024
***176***	26	Female	Kabul	Ibn-e-Sina	Lung	2024
***177***	26	Female	Kabul	Ibn-e-Sina	Lung	2025
***178***	26	Male	Takhar	Ibn-e-Sina	Lung	2025
***179***	26	Male	Kabul	Kabul university of medical sciences	Lung	2023
***180***	27	Male	Kabul	Esteghlal Hospital	Liver	2025
***181***	27	Female	Kabul	Ibn-e-Sina	Lung	2024
***182***	27	Female	Faryab	Ibn-e-Sina	Lung	2024
***183***	27	Female	Panjshir	Kabul university of medical sciences	Lung	2025
***184***	27	Male	Kabul	Ibn-e-Sina	Lung	2024
***185***	27	Male	Balkh	Ibn-e-Sina	Lung	2024
***186***	27	Male	Paktya	Ibn-e-Sina	Lung	2024
***187***	27	Male	Kabul	Ibn-e-Sina	Lung	2024
***188***	27	Male	Badakhshan	Ibn-e-Sina	Lung	2025
***189***	27	Male	Parwan	Ibn-e-Sina	Lung	2025
***190***	27	Male	Badakhshan	Ibn-e-Sina	Lung	2025
***191***	27	Male	Kabul	Ibn-e-Sina	Lung	2025
***192***	28	Female	Faryab	Ibn-e-Sina	Lung	2024
***193***	28	Female	Ghazni	Ibn-e-Sina	Lung	2025
***194***	28	Male	Takhar	Ibn-e-Sina	Lung	2024
***195***	28	Male	Faryab	Ibn-e-Sina	Lung	2024
***196***	28	Male	Kabul	Ibn-e-Sina	Lung	2025
***197***	29	Male	Konduz	Kabul university of medical sciences	Liver	2022
***198***	30	Female	Kunar	Esteghlal Hospital	Liver	2025
***199***	30	Female	Nangarhar	Ibn-e-Sina	Lung	2024
***200***	30	Female	Kandahar	Ibn-e-Sina	Lung	2024
***201***	30	Female	Konduz	Ibn-e-Sina	Lung	2024
***202***	30	Female	Kabul	Ibn-e-Sina	Lung	2024
***203***	30	Female	Balkh	Ibn-e-Sina	Lung	2024
***204***	30	Female	Balkh	Ibn-e-Sina	Lung	2025
***205***	30	Female	Baghlan	Ibn-e-Sina	Lung	2025
***206***	30	Male	Maydan Wardak	Ibn-e-Sina	Lung	2024
***207***	30	Male	Kapisa	Ibn-e-Sina	Lung	2024
***208***	30	Male	Kapisa	Ibn-e-Sina	Lung	2024
***209***	30	Male	Kabul	Ibn-e-Sina	Lung	2025
***210***	30	Male	Kabul	Ibn-e-Sina	Lung	2025
***211***	30	Male	Kabul	Ibn-e-Sina	Lung	2025
***212***	30	Male	Takhar	Ibn-e-Sina	Lung	2025
***213***	30	Male	Faryab	Ibn-e-Sina	Lung	2025
***214***	31	Male	Ghazni	Ibn-e-Sina	Lung	2024
***215***	31	Male	Kandahar	Ibn-e-Sina	Lung	2024
***216***	32	Female	Samangan	Ibn-e-Sina	Lung	2024
***217***	32	Female	Ghor	Ibn-e-Sina	Lung	2024
***218***	32	Female	Maydan Wardak	Ibn-e-Sina	Lung	2024
***219***	32	Female	Kabul	Ibn-e-Sina	Lung	2025
***220***	32	Male	Badakhshan	Ibn-e-Sina	Lung	2024
***221***	32	Male	Ghor	Ibn-e-Sina	Lung	2025
***222***	32	Male	Kabul	Kabul university of medical sciences	Lung	2022
***223***	33	Male	Kabul	Ibn-e-Sina	Lung	2024
***224***	33	Male	Kabul	Ibn-e-Sina	Lung	2025
***225***	33	Male	Faryab	Ibn-e-Sina	Lung	2025
***226***	33	Male	Konduz	Ibn-e-Sina	Lung	2025
***227***	33	Male	Khost	Ibn-e-Sina	Lung	2025
***228***	33	Male	Kabul	Ibn-e-Sina	Lung	2025
***229***	34	Male	Kabul	Ibn-e-Sina	Lung	2024
***230***	34	Male	Nangarhar	Ibn-e-Sina	Lung	2025
***231***	34	Male	Kabul	Ibn-e-Sina	Lung	2025
***232***	35	Female	Parwan	Esteghlal Hospital	Liver	2025
***233***	35	Female	Kabul	Esteghlal Hospital	Liver	2025
***234***	35	Male	Parwan	Kabul university of medical sciences	Liver	2022
***235***	35	Female	Maydan Wardak	Ibn-e-Sina	Lung	2024
***236***	35	Female	Kabul	Ibn-e-Sina	Lung	2024
***237***	35	Female	Kabul	Ibn-e-Sina	Lung	2025
***238***	35	Female	Kabul	Ibn-e-Sina	Lung	2024
***239***	35	Female	Baghlan	Ibn-e-Sina	Lung	2025
***240***	35	Male	Kabul	Ibn-e-Sina	Lung	2024
***241***	35	Male	Kabul	Ibn-e-Sina	Lung	2024
***242***	35	Male	Takhar	Ibn-e-Sina	Lung	2025
***243***	35	Male	Bamyan	Ibn-e-Sina	Lung	2025
***244***	35	Male	Sar e Pol	Kabul university of medical sciences	Lung	2022
***245***	36	Female	Baghlan	Ibn-e-Sina	Lung	2024
***246***	36	Female	Kabul	Ibn-e-Sina	Lung	2024
***247***	36	Female	Kabul	Ibn-e-Sina	Lung	2025
***248***	36	Male	Kabul	Ibn-e-Sina	Lung	2024
***249***	36	Male	Badakhshan	Ibn-e-Sina	Lung	2024
***250***	36	Male	Ghazni	Ibn-e-Sina	Lung	2025
***251***	38	Female	Kabul	Kabul university of medical sciences	Liver	2022
***252***	38	Female	Paktika	Kabul university of medical sciences	Lung	2021
***253***	39	Male	Kabul	Ibn-e-Sina	Lung	2025
***254***	39	Male	Herat	Kabul university of medical sciences	Lung	2022
***255***	40	Female	Kabul	Kabul university of medical sciences	Liver and Lung	2022
***256***	40	Female	Faryab	Ibn-e-Sina	Lung	2024
***257***	40	Female	Kabul	Ibn-e-Sina	Lung	2024
***258***	40	Female	Kabul	Ibn-e-Sina	Lung	2025
***259***	40	Female	Kapisa	Ibn-e-Sina	Lung	2025
***260***	40	Female	Balkh	Ibn-e-Sina	Lung	2024
***261***	40	Female	Konduz	Ibn-e-Sina	Lung	2024
***262***	40	Female	Kabul	Ibn-e-Sina	Lung	2024
***263***	40	Female	Badghis	Ibn-e-Sina	Lung	2025
***264***	40	Female	Badghis	Ibn-e-Sina	Lung	2025
***265***	40	Female	Kabul	Ibn-e-Sina	Lung	2025
***266***	40	Female	Baghlan	Ibn-e-Sina	Lung	2025
***267***	40	Male	Balkh	Ibn-e-Sina	Lung	2024
***268***	42	Female	Faryab	Kabul university of medical sciences	Liver	2022
***269***	42	Female	Ghazni	Ibn-e-Sina	Lung	2024
***270***	42	Female	Faryab	Ibn-e-Sina	Lung	2024
***271***	42	Male	Paktika	Ibn-e-Sina	Lung	2024
***272***	42	Male	Konduz	Ibn-e-Sina	Lung	2024
***273***	44	Female	Kabul	Kabul university of medical sciences	Lung	2023
***274***	45	Female	Bamyan	Ibn-e-Sina	Lung	2025
***275***	45	Female	Sar pol	Ibn-e-Sina	Lung	2024
***276***	45	Female	Faryab	Ibn-e-Sina	Lung	2024
***277***	45	Female	Faryab	Ibn-e-Sina	Lung	2025
***278***	45	Male	Paktya	Ibn-e-Sina	Lung	2025
***279***	46	Male	Kabul	Kabul university of medical sciences	Liver	2023
***280***	46	Female	Badghis	Ibn-e-Sina	Lung	2024
***281***	47	Male	Takhar	Ibn-e-Sina	Lung	2024
***282***	47	Male	Balkh	Ibn-e-Sina	Lung	2024
***283***	47	Male	Kabul	Ibn-e-Sina	Lung	2024
***284***	48	Male	Balkh	Ibn-e-Sina	Lung	2024
***285***	48	Male	Takhar	Ibn-e-Sina	Lung	2024
***286***	48	Male	Samangan	Ibn-e-Sina	Lung	2025
***287***	48	Male	Kabul	Ibn-e-Sina	Lung	2025
***288***	49	Female	Kabul	Ibn-e-Sina	Lung	2025
***289***	49	Male	Samangan	Ibn-e-Sina	Lung	2025
***290***	50	Female	Samangan	Esteghlal Hospital	Liver	2025
***291***	50	Female	Kabul	Ibn-e-Sina	Lung	2025
***292***	50	Female	Kabul	Ibn-e-Sina	Lung	2025
***293***	50	Female	Faryab	Kabul university of medical sciences	Lung	2023
***294***	50	Male	Faryab	Ibn-e-Sina	Lung	2024
***295***	50	Male	Samangan	Ibn-e-Sina	Lung	2025
***296***	51	Male	Jawzjan	Esteghlal Hospital	Liver	2025
***297***	51	Male	Kabul	Ibn-e-Sina	Lung	2024
***298***	51	Male	Jawzjan	Ibn-e-Sina	Lung	2025
***299***	52	Female	Parwan	Ibn-e-Sina	Lung	2024
***300***	53	Female	Badakhshan	Ibn-e-Sina	Lung	2025
***301***	53	Female	Takhar	Kabul university of medical sciences	Lung	2021
***302***	53	Male	Jawzjan	Ibn-e-Sina	Lung	2024
***303***	55	Female	Takhar	Ibn-e-Sina	Lung	2025
***304***	55	Female	Badghis	Ibn-e-Sina	Lung	2024
***305***	55	Male	Jawzjan	Ibn-e-Sina	Lung	2024
***306***	55	Male	Faryab	Ibn-e-Sina	Lung	2025
***307***	55	Male	Ghor	Ibn-e-Sina	Lung	2025
***308***	56	Female	Takhar	Ibn-e-Sina	Lung	2024
***309***	56	Female	Kabul	Ibn-e-Sina	Lung	2025
***310***	58	Female	Faryab	Kabul university of medical sciences	Lung	2023
***311***	58	Male	Kabul	Ibn-e-Sina	Lung	2024
***312***	58	Male	Faryab	Ibn-e-Sina	Lung	2025
***313***	60	Female	Kabul	Esteghlal Hospital	Liver	2025
***314***	60	Female	Konduz	Ibn-e-Sina	Lung	2025
***315***	60	Female	Faryab	Ibn-e-Sina	Lung	2025
***316***	60	Female	Paktya	Ibn-e-Sina	Lung	2025
***317***	60	Male	Kabul	Ibn-e-Sina	Lung	2024
***318***	60	Male	Takhar	Ibn-e-Sina	Lung	2025
***319***	60	Male	Konduz	Ibn-e-Sina	Lung	2025
***320***	63	Male	Faryab	Ibn-e-Sina	Lung	2024
***321***	64	Male	Faryab	Ibn-e-Sina	Lung	2025
***322***	65	Male	Faryab	Kabul university of medical sciences	Liver and Lung	2022
***323***	65	Male	Takhar	Ibn-e-Sina	Lung	2025
***324***	67	Male	Kabul	Ibn-e-Sina	Lung	2025
***325***	67	Male	Ghor	Kabul university of medical sciences	Lung	2025
***326***	70	Female	Bamyan	Esteghlal Hospital	Liver	2025
***327***	70	Female	Takhar	Ibn-e-Sina	Lung	2024
***328***	75	Male	Kabul	Ibn-e-Sina	Lung	2025
***329***	78	Female	Kabul	Ibn-e-Sina	Lung	2025
***330***	20	Female	Balkh	Refah Hospital	Brain	2025

* Patients in the thoracic and cardiovascular surgery department of Kabul University of Medical Sciences have been diagnosed, operated on, and treated.

**Fig 1:**
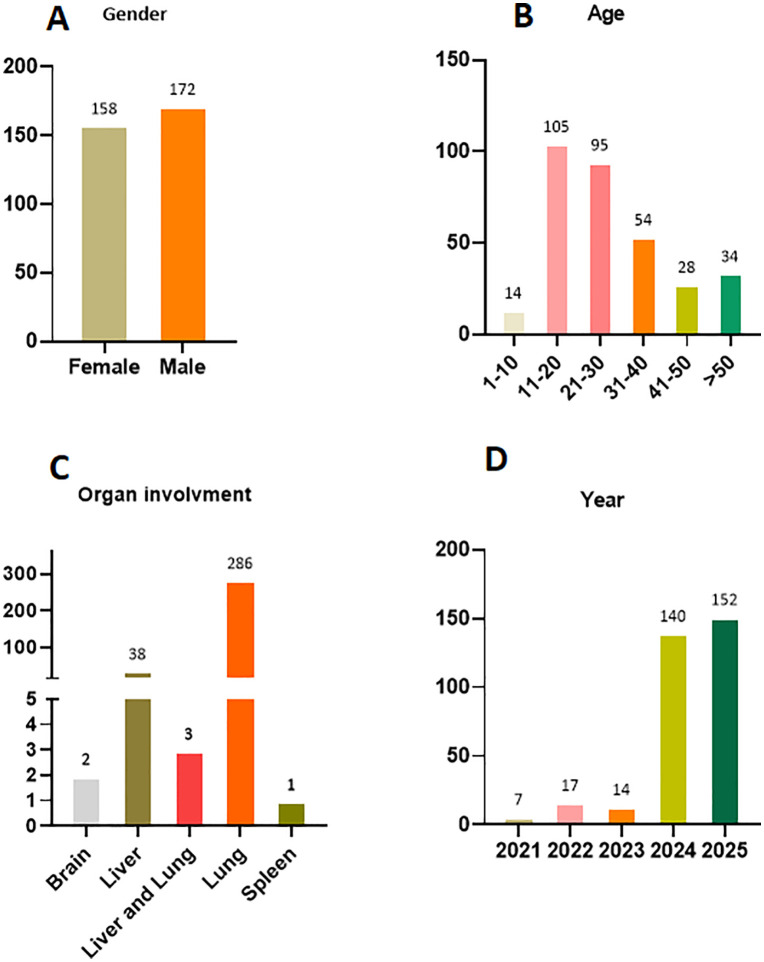
Gender, age group, temporal data, and organ involvement distribution of CE patients admitted to five referral hospitals in Kabul, Afghanistan (2021–2025). A: Shows the gender breakdown; B: illustrates the age group distribution; C: details affected organs; D: Shows temporal (year) data.

Regarding gender distribution, there was no significant male predominance; 172 patients (52.12%) were male, while 158 patients (47.87%) were female. The age range was 6–78 years, with 91 patients (27.57%) under 20 years (Fig 1). When interpreted in the context of Afghanistan’s population demographics, where roughly 43% are under 15 years, one might expect a disproportionately higher number of CE cases in children if all age groups were equally susceptible.

**Fig 2:**
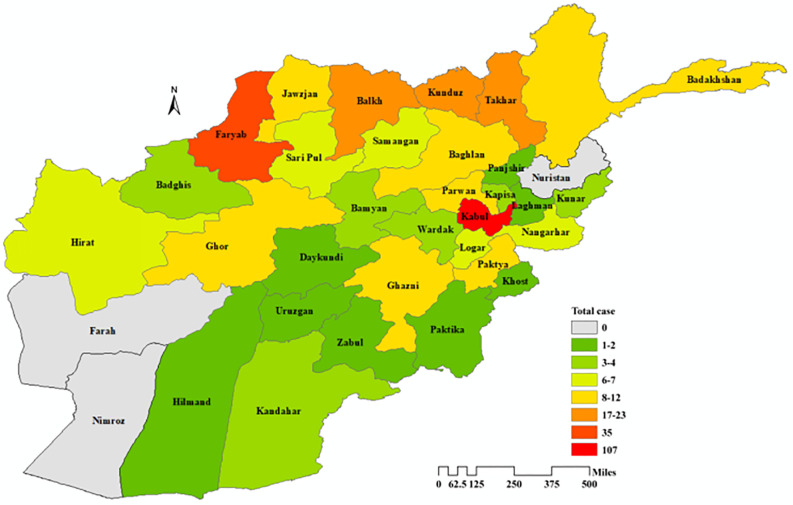
Geographic distribution of CE cases, derived from hospital records. The intensity of color in each province is proportional to the total number of CE cases reported for that region. This map was created using ArcGIS software and the source of the base map shapefile on which the data is plotted is available at this link https://geo.btaa.org/catalog/stanford-zr035kz3919.

In terms of geographic distribution, patients were recruited from 31 provinces (Fig 2). Kabul province had the highest number of cases (107 cases), making it the primary region of origin for study participants. Faryab and Balkh Provinces followed as the next most common regions of origin (35 and 24 cases respectively). Patients were referred to Kabul’s five referral hospitals from 31 provinces across Afghanistan due to limited diagnostic, imaging, and surgical capacity in provincial healthcare facilities. All included patients required and received surgical intervention for CE. The remaining provinces each contributed between 1 and several CE cases, reflecting a scattered geographic distribution of the disease across these regions. In contrast, no cases were referred from Farah, Nimroz, and Nuristan, which may reflect limited healthcare access rather than absence of disease.

Among the 330 patients diagnosed with CE, the lungs were the most frequently affected organ, accounting for 86.66% of cases (286/330). Up to 81.31% of patients aged under 20 presented with pulmonary cysts. The liver was the second most commonly involved organ, representing 11.51% of cases (38/330). The high proportion of pulmonary CE is expected and not remarkable, as pulmonary CE becomes symptomatic earlier than hepatic CE, leading to earlier hospital presentation. Similarly, the higher proportion of young patients is also explained by this earlier symptomatic presentation.

A total of two patient (0.6%) had isolated brain CE and one spleen CE (0.3%), with no concurrent involvement of other organs. Additionally, three patients (0.9%) presented with CE affecting both the liver and lungs.

A total of 130 patients with lung CE were female (45.45%; 130/286). Younger patients (<20 years) were more likely to have lung involvement (74/286, 25.87%) compared to older patients (212/286; 74.12%). 130 of 158 women (82.27%) had pulmonary CE, while 156 of 172 men (90.62%) had pulmonary CE.

Analysis of the yearly distribution of CE cases revealed that, 150 of 330 CE cases (45.45%) were recorded in 2025, while the combined total for 2021–2024 was 180/330 (54.54%), suggesting this likely reflects improved record (implementation of a standardized paper-based archiving system) retrieval rather than a true increase in incidence.

## Discussion

The most striking finding of this study is the extremely high proportion of pulmonary CE (86.66%), representing the dominant clinical presentation among surgically treated patients in Kabul. This differs markedly from global patterns where hepatic cysts are most common, and underscores the unique healthcare seeking and clinical presentation profile in this setting.

CE remains a major NTD in endemic regions, including the Tibetan, Pamir, and Iranian Plateaus, the broader Middle East, Central Asia, and parts of Africa and South America [3,18,19]. The disease imposes substantial public health and economic burdens through healthcare costs, productivity losses, and livestock-related impacts [12,20,21].

Although population-based data are lacking in Afghanistan, the country lies within a highly endemic Central Asian region, placing millions at risk, [22]. High endemicity is driven by fragile health governance, poverty, political instability, limited healthcare access, close human–animal contact, and livestock-dependent livelihoods, particularly in rural populations [22–24].

Similar challenges affect neighboring countries. In Pakistan, the true burden of CE remains underestimated due to absent national surveillance and fragmented reporting [19]. In Iran, reported incidence likely underrepresents true prevalence because many infections are asymptomatic and unreported [25].

In our study, geographically, the enrolled patients were recruited from 31 province (Fig 2). All study patients underwent surgery in Kabul, as medical record reviews confirmed many were referred from their home provinces, specifically due to insufficient local facilities. This distribution likely reflects both Kabul’s high population density and the role of livestock-dependent transmission in rural provinces, suggesting the complex interplay between urban and rural epidemiological factors. It is worth noting that no cases were identified from Farah, Nimroz and Nuristan. This absence is likely due to barriers in healthcare access rather than lower disease prevalence, as a prior study conducted in Khost documented a CE prevalence of 1.75% among inpatients [25]. As highlighted by Tamarozzi et al. (2018), hospital-based records may underestimate the true prevalence of abdominal CE by up to 700-fold in some endemic regions, such as rural Romania, underscoring that inpatient data alone cannot fully capture the actual spread of CE, even in settings where cases are formally documented [26].

Our findings indicate that hospital-reported CE cases reached their highest proportion in 2025 (45.45%), highlighting the apparent urgency for targeted public health interventions. However, this peak should not be interpreted as a true increase in disease incidence during that year. Afghanistan lacks a centralized Hospital Information System (HIS), and all available data are derived from paper-based records. As a result, retrieving patient information from previous years is extremely challenging. Consequently, the diagnosed cases likely represent only a small fraction of the actual disease burden in the country.

The demographic distribution of cases should also be interpreted with caution. Although the highest number of cases was observed in the 10–20-year age group, this finding does not necessarily indicate increased susceptibility or exposure among younger individuals. Afghanistan has a predominantly young population, with approximately 43% under the age of 15 [27]; therefore, a higher absolute number of cases among younger age groups would be expected. To determine whether age is truly associated with disease risk, age-stratified incidence rates would be required. It is plausible that older individuals may face a higher risk; however, the smaller number of observed cases in these age groups may simply reflect their lower population size rather than a reduced susceptibility.

This age distribution aligns with regional patterns in Central Asia, where high pediatric CE cases correlate with unregulated livestock slaughter, stray dog populations, and limited veterinary oversight all drivers that sustain *E. granulosus* transmission [28]. The predominance of pulmonary cysts (81.31%) in these young patients further supports active transmission, as larval migration to the lungs is a common outcome of recent infection in younger hosts. Collectively, the high proportion of under-20 cases confirms that *E. granulosus*’s zoonotic cycle is functional and unchecked in Afghanistan, with continuous spillover to human populations.

In this study, 47.87% of patients were female, indicating no significant gender predominance. This gender distribution contrasts with findings from other studies, which often report a higher number of cases among women than men. In those settings, women’s routine involvement in household and agricultural activities is thought to increase their exposure to the parasite [29,30].

Several factors may explain the pattern observed in our study and warrant further discussion. In Afghanistan, women constitute approximately 33% of the workforce in agriculture and related sectors, with rural women primarily engaged in small-scale dairy farming. Historically, their roles have been concentrated on milk production, highlighting their importance within local agricultural systems [31–35]. As CE is more prevalent in rural, agriculture-dependent communities, lower female participation in agricultural activities compared with men may result in reduced exposure, potentially explaining the lower proportion of female cases observed in our data. Another contributing factor may be the social exclusion of Afghan women from many aspects of public life. If such exclusion extends to inequitable access to healthcare services, infected women may be less likely to seek or receive diagnosis and treatment than men. This would lead to an underrepresentation of women among treated and reported cases, despite the possibility that the true burden of untreated infection among women may be greater than that among men.

We observed a marked predominance of pulmonary CE in this cohort, with pulmonary involvement accounting for approximately 87% of cases. The predominance of pulmonary CE and the higher proportion of young patients are expected findings, because pulmonary CE manifests symptoms earlier than hepatic CE, leading to earlier hospital admission and treatment [1]. In addition, a large proportion of patients were young, with 81.31% (74/91) under 20 years of age. This pattern is highly unusual, as most published studies report hepatic involvement as the dominant presentation, with pulmonary CE accounting for only around 20% of cases [3].

This striking deviation from the expected organ distribution warrants careful consideration. One plausible explanation is a bias related to healthcare-seeking behavior and case ascertainment. Pulmonary echinococcosis is generally more symptomatic and clinically severe than hepatic disease, which may prompt affected individuals to seek medical care. In contrast, hepatic echinococcosis is frequently asymptomatic or relatively benign, particularly in early stages, and may therefore remain undiagnosed and untreated. As a result, hospital-based data may disproportionately capture pulmonary cases, leading to an overrepresentation of lung involvement among treated patients.

This pattern may be further compounded by limited health infrastructure; whereby medical services are effectively rationed and only the most severely affected individuals receive treatment. Alternatively, the observed predominance of pulmonary disease may reflect the *Echinococcus* genotype circulating in Afghanistan. To our knowledge, no studies have investigated *Echinococcus* genotypes in this setting; however, elsewhere the G6 genotype has been associated with a higher frequency of pulmonary echinococcosis, warranting further investigation.

The age distribution of lung cysts is also notable, with younger individuals showing an even greater predominance of pulmonary involvement. This contrasts with earlier findings, including a study conducted over two decades ago, which reported hepatic cysts to be more common among younger patients [28].

Evidence from northern Afghanistan further supports ongoing transmission, with radiological and serological screening identifying cysts in multiple organs among high-risk populations [15].

Reports from the region and elsewhere have documented CE in organs beyond the liver and lungs, including the orbit, pleura, spinal cord, brain, muscles, uterus, pancreas, and spleen [13,14,36–51]. In the absence of robust national surveillance data, these findings contribute important insights into the epidemiology of CE in Afghanistan and underscore the need for comprehensive diagnostic vigilance in endemic settings. Without a national registry or functional monitoring system, the true disease burden remains largely hidden and is inferred mainly from retrospective hospital data and limited local surveys, which substantially underestimate prevalence due to asymptomatic infections, diagnostic gaps, geographic bias, and fragmented reporting. CE’s long asymptomatic course and limited access to diagnostic imaging in livestock-dependent rural areas further contribute to underdiagnosis. Evidence of this hidden burden is illustrated by a serosurvey in Sheberghan, northern Afghanistan, where 10.2% of household contacts were seropositive and 5.1% had radiological cysts, far exceeding hospital-based estimates [15]. Hospital-based data in Afghanistan likely overrepresent urban and peri-urban populations, while rural provinces with high livestock exposure and understudied southern regions remain largely unexamined. Additionally, existing hospital studies often record treatment or referral locations rather than patients’ true origins, further biasing geographic patterns. Robust surveillance and a national disease registry are therefore essential to accurately characterize the epidemiology of echinococcosis in Afghanistan.

The burden of CE in Afghanistan is exacerbated by large stray dog populations, the primary definitive host of *E. granulosus*. A landmark 1988 post-mortem survey in Kabul found 73.3% of stray dogs infected, with parasite loads reaching up to 152,700 worms and infections detected in dogs as young as four months, indicating intense and early transmission [52].

Despite the absence of recent data, these findings remain relevant given the lack of mass deworming and widespread unregulated slaughter. High stray-to-owned dog ratios and frequent dog access to livestock offal sustain environmental contamination and ongoing transmission [53]. Together with weak surveillance, limited healthcare access, poverty, and livestock-dependent livelihoods, these factors entrench CE as a neglected public health issue that disproportionately affects vulnerable populations, including children, women in agricultural and household roles, and rural communities.

Interrupting the dog–livestock lifecycle of *E*. *granulosus* necessitates integrated One Health interventions, yet such measures are largely absent in Afghanistan. Community awareness of CE transmission is minimal, no national hygiene or prevention campaigns exist, and community health workers receive no CE-specific training. Despite outdated data, stray dogs, particularly in Kabul, likely remain a major reservoir [52] due to the absence of mass deworming and control programs. Unregulated slaughter practices, unhygienic abattoirs, unrestricted dog access to infected offal, and severely limited veterinary public health capacity further sustain transmission [54].

Access to CE diagnosis and treatment in Afghanistan is severely limited. The WHO 2021–2030 NTD Roadmap emphasizes equitable access to care through health-system integration, yet protracted conflict, fragile governance, poverty, climate change, and persistent deficits in healthcare, safe water, sanitation, and housing continue to undermine these goals [55,56]. Surgical capacity is restricted to a few centers, and underdiagnosis remains common, particularly in rural livestock-dependent communities.

Human CE transmission occurs through contact with infected dogs and exposure to contaminated soil, water, and possibly food [57–63]. Free-roaming dogs contaminate surface water and produce, making raw or unwashed vegetables a key risk factor [57,59], while *E. granulosus* ranks among the leading foodborne parasites globally [2,64]. Although contaminated water is increasingly recognized as a transmission route [2], environmental evidence remains limited [58,65], and is entirely lacking in Afghanistan. Widespread lack of piped water (>80% of households) [66] and poor sanitation, particularly in slums or urban informal settlements (five million people; 75%), further increase exposure risk [67].

In Afghanistan, severe food insecurity, affecting over 11.6 million people in late 2024 and projected to worsen in 2025, compounds reliance on unsafe water and poorly washed food, amplifying CE transmission risk [68,69]. This convergence of unsafe water, inadequate sanitation, and food insecurity increases reliance on contaminated water sources and poorly washed produce, amplifying potential CE exposure.

**Fig 3:**
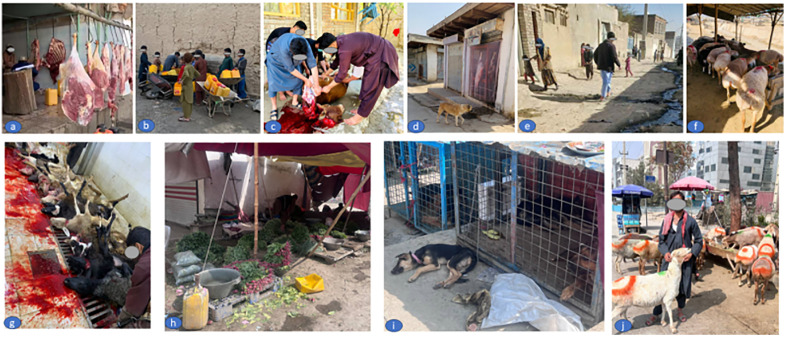
Multidimensional risk factors driving CE transmission in Afghanistan. Key interrelated factors perpetuating zoonotic transmission of *E. granulosus* in Afghanistan’s fragile context: (a) poor environmental hygiene in animal product retail outlets; (b) limited access to safe drinking water; (c) unregulated home-based slaughter facilities; (d) uncontrolled free-roaming dog populations in urban areas; (e) socioeconomic deprivation and poverty; (f) traditional livestock husbandry practices; (g) unregulated commercial abattoirs; (h) street vending of vegetables (i) widespread dog trading outlets; (j) cohabitation of livestock with human residences. These factors interact to sustain the CE transmission cycle and exacerbate health inequities. All photographs in Fig 3 were taken by the authors, with consent for publication under CC BY 4.0 license.

These systemic failures, ranging from limited surveillance to unequal treatment access, intersect with environmental, behavioral, and socioeconomic factors to sustain CE transmission in Afghanistan. As summarized in Fig 3, poor sanitation, unregulated animal practices, close human–livestock–dog contact, and poverty collectively reinforce human exposure to *E. granulosus*. Many of these risks, including home slaughter (Fig 3c) and reliance on unsafe water (Fig 3b), are rooted in poverty and fragile livelihoods, creating a self-reinforcing cycle in which CE both results from and exacerbates economic vulnerability among Afghan households.

CE reflects and reinforces poverty in Afghanistan, one of the world’s poorest countries, where over 40% of the population lives below the national poverty line [70] and around 70% depends on subsistence livestock farming, conditions that directly facilitate zoonotic transmission [24]. Regional WHO EMRO estimates suggest that between 14.0 and 16.2 million people in Afghanistan required NTD interventions between 2012 and 2019, although these figures derive from regional models rather than national prevalence surveys, highlighting major data gaps [71]. The association between poverty and NTDs is well established [72], with infectious diseases disproportionately affecting poor and marginalized populations and perpetuating a cycle of ill-health and economic deprivation [73]. NTDs, including CE, thrive in contexts of poor sanitation, limited healthcare access, weak veterinary and public-health systems, and close human–animal contact [74–79], all of which are strongly linked to poverty. In turn, chronic illness, disability, stigma, and productivity losses impair education and livelihoods, trapping households in long-term poverty [73,75], particularly in livestock-dependent communities where inadequate veterinary services and unsafe slaughter practices are common. Globally, over 600 million people depend on livestock for livelihoods, facing both zoonotic risks and indirect losses from reduced animal productivity [80], while social and political marginalization further limits access to prevention and care [73]. In Afghanistan, conflict, political instability, population displacement, food insecurity, and the erosion of health and veterinary infrastructure exacerbate these dynamics, increasing vulnerability to NTDs [72,81,82]. Although contemporary nationwide CE prevalence data are scarce, historical surveys and case reports indicate ongoing transmission. Evidence from LMICs shows that CE imposes substantial direct and indirect economic costs, including medical expenses, livestock productivity losses, and reduced human productivity [12,16], disproportionately affecting poor rural households and reinforcing a poverty–disease cycle [83]. In Afghanistan, surgical treatment costs can equal a year’s income for rural farmers, while long-term disability, lost labor, child labor, educational disruption, and livestock production losses deepen intergenerational poverty. Breaking this self-reinforcing cycle requires coordinated, multisectoral One Health approaches that integrate CE control with broader NTD, WASH, primary healthcare, and humanitarian services [84,85], supported by good governance, political commitment, sustained funding, community engagement [86], and long-term investments in resilient health and veterinary systems and international partnerships [87–89]. To effectively address the persistent burden of CE in Afghanistan and break the interconnected cycle of poverty and disease, a coordinated, multisectoral approach rooted in the One Health principle is imperative, with key actionable strategies detailed in Table 2.

**Table 2:** Urgent need for action, a multisectoral one health framework for CE control in Afghanistan.

Section	Subsection	Details	Prerequisites/Adaptations
1. Establish Nationwide CE Surveillance to Map Burden and Trends	-	-	Stabilize hospital operations with basic electricity, supplies, and digitization tools; bolster CHW networks for rural reach to enable effective data collection.
	1.1 Mandatory Centralized Hospital Registry	The Ministry of Public Health (MoPH) should mandate CE case reporting in all tertiary and provincial hospitals, with standardized data fields: - Demographics (age, sex/gender, province of origin) - Clinical details (cyst location, recurrence status, treatment type) Data should be digitized via the existing Hospital Information System (HIS) to enable real-time monitoring, mapping of endemic foci, and tracking of rare organ localizations.	Start with pilots in major cities like Kabul to test feasibility before full rollout.
	1.2 Targeted Community Screening	Deploy mobile diagnostic units to rural areas for serological (ELISA) and ultrasound screening of high-risk groups, including: A. Household contacts of confirmed CE patients (elevated exposure risk). B. Livestock farmers and their households (frequent dog/livestock contact). C. Rural children aged 5–15 years (immature immune systems + increased contact with contaminated soil/food). Integrate screening into existing public health campaigns (e.g., polio vaccination drives) to leverage community health worker (CHW) networks and minimize costs.	Piggyback on ongoing ICRC or WHO primary care efforts for initial rollout in accessible areas.
2. Strengthen Diagnostic and Treatment Capacity for Equitable Care	-	-	Secure international donations for equipment and supplies; align with the Health Sector Transition Strategy (2023–2025) or its successors for service quality improvements, focusing on emergency response and rural access.
	Equipment and Workforce Training	Equip rural hospitals with ultrasound machines (for cyst detection) and train clinicians in CE diagnosis (e.g., WHO classification) and management (e.g., surgical and medical therapy or watch and wait).	Begin with short-term NGO-led training programs to build capacity quickly.
	Ensure Medicine Access	Stockpile anti-CE medications (e.g., albendazole) in rural health facilities to avoid treatment delays.	Partner with global health initiatives for consistent imports and supply chain stability.
	Specialized Referral Centers	Establish CE-focused referral centers in regional hubs to manage complex cases.	Build on existing urban facilities to minimize new infrastructure needs.
	Teleconsultation Services	Provide rural hospitals with teleconsultation support from specialists to guide diagnostic and treatment decisions for complex cases.	Use low-tech alternatives like radio if internet is unreliable in remote areas.
3. Scale Prevention and Control to Break CE Transmission Cycles	-	-	Strengthen animal health infrastructure through community animal health workers (CAHWs); address food security shocks and poverty to support community compliance and reduce reliance on informal practices.
	3.1 Veterinary and Animal-Related Interventions	-	-
		Mass Dog Deworming: Implement regular deworming programs for stray and owned dogs, with community engagement to ensure compliance.	Train CAHWs for hard-to-reach areas, starting with community-led pilots.
		Slaughter Regulation: Standardize hygiene practices in abattoirs and restrict home slaughter by providing small grants to poor households for access to hygienic commercial slaughter.	Address poverty barriers first through integrated aid programs to ensure grants are effective.
		Sheep Vaccination: Pilot the EG95 vaccine in high-endemic provinces to interrupt the parasite lifecycle.	Ensure cold chains and basic vet oversight are in place before piloting.
	3.2 Community-Focused Health Education	Deliver culturally tailored messages via trusted local channels: - Community radio broadcasts - Announcements at religious sites (e.g., mosques) - Home visits by female CHWs Emphasize actionable behaviors: - Wash vegetables with safe water to remove parasite eggs. Sanitize hands after contact with animal or soil. Distribute pictorial leaflets (for low-literacy populations) at health centers, schools, and markets, highlighting CE symptoms, transmission routes, and preventive steps.	This can be implemented early as a low-cost entry point, leveraging existing local networks without advanced infrastructure.
4. Invest in Context-Specific Research to Inform Evidence-Based Policy	-	-	Collaborate with international bodies for funding and expertise; integrate with broader health surveys to share costs and avoid standalone resource drains.
	1. National CE Seroprevalence Survey	Map geographic hotspots, at-risk populations, and animal reservoir distribution to target resources.	Start with limited-scope surveys in priority provinces.
	2. Minimally Invasive Treatment Evaluation	Assess the safety, efficacy, and cost-effectiveness of treatments like PAIR (Percutaneous Aspiration, Injection, Reaspiration) in resource-limited settings.	Pilot in urban centers with existing medical support.
	3. Transmission Cycle Research	Investigate E. granulosus spillover dynamics between animal hosts and humans, plus environmental persistence of parasite eggs, to identify key transmission bottlenecks.	Focus on collaborative studies with minimal fieldwork requirements initially.

This study has limitations stemming from its retrospective, hospital based surgical design. As a case series limited to symptomatic, surgically treated patients from Kabul’s referral hospitals, our findings provide minimal generalizable epidemiological insights and primarily reflect the characteristics of patients who accessed specialized surgical care. Our descriptive data cannot distinguish between true population level disease patterns and inequalities in healthcare access, and selection bias limits our ability to generalize findings to the overall prevalence or epidemiological profile of CE in Afghanistan. Notably, the high proportion of pulmonary cases likely reflects earlier symptomatic presentation and healthcare seeking behavior rather than the actual population-level distribution of CE.

### Concluding remarks

Cystic Echinococcosis remains a pressing public health challenge in Afghanistan, deeply intertwined with the country’s socioeconomic realities, livestock-dependent livelihoods, and fragile health infrastructure.

To effectively mitigate CE’s impact, a coordinated, multisectoral One Health approach is imperative. This must include strengthening nationwide surveillance through centralized case reporting and targeted community screening, expanding diagnostic and treatment capacity in rural areas, and scaling preventive interventions such as mass dog deworming, regulated slaughter practices, and culturally tailored health education. Additionally, investing in context-specific research including investigating local *Echinococcus* genotypes and evaluating minimally invasive treatment options will inform evidence-based policy and program design.

As Afghanistan navigates ongoing challenges of political instability, food insecurity, and health system fragility, addressing CE requires sustained commitment from national authorities, international partners, and local communities. By integrating CE control into broader public health, veterinary, and development agendas, it is possible to break the transmission cycle, reduce health inequities, and alleviate the dual burden of disease and poverty. Although this study is limited to a sample of surgically treated patients in Kabul, it emphasizes the importance of monitoring and managing this disease to prevent serious consequences for the community.

## Methods

### Ethics statement

This study was reviewed by the Ghalib University Institutional Review Board (GUIRB) and approved by the Ethics Committee in Biomedical Researches (AF.GUIRB.ECBR.1404.001). Formal ethical approval was granted for the retrospective analysis of de-identified patient medical records, in accordance with the committee’s guidelines for observational hospital-based research in Afghanistan.

Written informed consent was not obtained from study participants for this retrospective study, as all data analyzed were extracted from de-identified, archived medical records with all personal identifying information (e.g., names, contact details, medical record numbers) removed prior to data collection. The Afghanistan Research Ethical Committee waived the requirement for informed consent due to the anonymized nature of the data and the minimal risk to participants associated with this retrospective analysis. Strict measures were implemented throughout the research process to protect patient privacy and comply with ethical protocols for human research subject data in Afghanistan.

### Study area

Kabul serves as both the national capital of Afghanistan and the capital of Kabul Province, and it also functions as one of the province’s administrative districts. As the country’s largest urban center, it stands as Afghanistan’s core political and economic hub, located in the eastern part of the nation at latitude 34.5553° North and longitude 69.2075° East. Nestled in an east-central high-altitude basin, at approximately 5,900 feet (1,800 meters) above sea level. The city lies in a triangular valley between mountain ranges, covering an area of 400 square miles (1,030 square kilometers) and boasting an estimated 2017 population of 3,961,500. Kabul experiences a semi-arid continental climate, with rainfall primarily in spring and early August; summers average around 90 °F (32 °C) with typical around 20 °C diurnal (day-night) temperature differences, while winters average 20 °F.

### Data collection

This retrospective study was conducted on patients admitted to the medical and surgical wards of five referral hospitals in Kabul Province, Afghanistan, including Maiwand pediatic, Refah, Esteghlal, Ibn-e-Sina and thoracic and cardiovascular surgery hospital, from 2021 to 2025. Data were gathered from archived hospital medical records retrieved via the archived medical documents. Patient origin was defined as the self-reported permanent residential province, extracted from admission forms and clinical notes in archived medical records. Variables assessed included demographic information (age, sex, residential area), and affected organs. For the analysis, descriptive results were prioritized in the interpretation.

In Afghanistan, CE is not a nationally notifiable disease. Patients were referred to Kabul’s hospitals from provincial health centers due to limited diagnostic and surgical capacity outside the capital. Human CE were diagnosed using chest X‑ray, ultrasound and computed tomography (CT). In this study patient data were retrospectively extracted from archived paper medical records; a semi-standardized archiving system was implemented in 2025, improving case retrieval. Historically, Afghanistan has not had a national CE surveillance or control program in humans or animals. This study only includes patients who received surgical treatment at referral hospitals, which limits epidemiological interpretation and may reflect healthcare access rather than actual disease burden.

### Data and materials

All underlying data supporting the findings of this study are fully presented in Table 1 of the manuscript (Demographic, regional, institutional/hospital, and temporal distribution of 330 CE cases). This table includes all de-identified patient-level data analyzed for the research: age, gender, provincial origin, treating hospital, affected organ, and year of admission. No additional raw data were generated or used beyond the information in Table 1, and the de-identified aggregate data (e.g., gender/organ/age group distributions) reported in the Results section (and presented in the manuscript’s text and Fig 1) are derived exclusively from this table.
